# Climate change and ocular health: temperature-pollution synergies amplify uveitis burden

**DOI:** 10.3389/fpubh.2025.1650255

**Published:** 2025-07-25

**Authors:** Yue Tan, Yue Yin, Boya Lei, Min Zhou, Zhengyue Gu, Jingjing You, Tong Lin, Lan Gong

**Affiliations:** ^1^Department of Ophthalmology, Eye Institute, Eye and ENT Hospital, Fudan University, Shanghai, China; ^2^NHC Key Laboratory of Myopia and Related Eye Diseases, Key Laboratory of Myopia and Related Eye Diseases, Chinese Academy of Medical Sciences, Shanghai, China; ^3^Shanghai Key Laboratory of Visual Impairment and Restoration, Shanghai, China; ^4^School of Medical Sciences, University of Sydney, Sydney, NSW, Australia

**Keywords:** uveitis, outpatient visits, temperature, distributed lag nonlinear model, air pollution

## Abstract

**Background:**

Uveitis, an inflammatory eye disease, exhibits seasonal patterns, which suggest environmental influences. This study examines the link between average temperature and uveitis outpatient visits, considering air pollution’s modifying effects.

**Methods:**

We analyzed uveitis outpatient data (*n* = 8,090) from a major hospital in Shanghai between 2017 and 2023, along with meteorological and air pollutant data. A distributed lag non-linear model (DLNM) was used to assess the associations between temperature and outpatient visits, adjusting for humidity, pollutants, and temporal factors.

**Results:**

A non-linear relationship exists between temperature and uveitis visits. Lower temperatures increased visits, with peak relative risk at −4°C lagged by 1 day (RR = 1.351, 95%CI: 1.069–1.706). Significant associations were found at lags 0–1 and 12–14, with the highest risk at lag 14 (−4°C, RR = 1.257, 95%CI: 1.113–1.420). Stratified analyses showed stronger associations in males and individuals under 60 years. High humidity and elevated PM_2.5_ levels strengthened the cold temperature association, while extremely high temperatures (33–34°C) increased visits under low humidity (RR = 2.625, 95%CI: 1.034–6.668 at 34°C).

**Conclusion:**

Temperature extremes are linked to increased uveitis outpatient visits in Shanghai, particularly with cold temperatures in high-humidity and high-PM_2.5_ environments, and hot temperatures under low humidity.

## Introduction

Uveitis encompasses a diverse group of inflammatory ocular disorders affecting the uveal tract (iris, ciliary body, and choroid) and potentially adjacent structures such as the retina and vitreous (1–4). This condition significantly impacts visual function, accounting for 5–10% of global visual impairment cases, and can manifest as an initial presentation of systemic infections or autoimmune diseases (3, 5–7). The global prevalence of uveitis ranges from 38–714 per 100,000 population, with annual incidence rates of 17–52 per 100,000, showing substantial regional and population variations (6, 8).

The heterogeneity of uveitis is reflected in its diverse clinical manifestations, etiologies, and anatomical distributions. The Standardization of Uveitis Nomenclature Working Group classifies uveitis into anterior, intermediate, posterior, and panuveitis categories based on the primary site of inflammation (9). Etiologically, uveitis may arise from infectious agents (viruses, bacteria, parasites) or non–infectious factors (autoimmune and systemic diseases) (10–12). The epidemiological characteristics vary significantly across regions, influenced by geographic location, ethnicity, genetic predisposition, environmental factors, and socioeconomic conditions (2, 13, 14). In developing countries, infectious uveitis predominates (15, 16), while non–infectious forms, particularly HLA–B27–associated anterior uveitis, are more common in developed nations (5, 17, 18).

Recent epidemiological evidence suggests that environmental factors may influence the occurrence of ocular inflammation related diseases (19, 20). Several studies have identified potential associations between meteorological conditions and uveitis incidence. Tan et al. reported a correlation between temperature increases and elevated uveitis incidence in mainland China (21), while other research has identified humidity and low temperatures as potential risk factors (22, 23). These findings suggest climate parameters might play a role in uveitis epidemiology.

Additionally, emerging evidence indicates potential links between air pollution and uveitis risk. A large Taiwanese cohort study revealed associations between air pollution exposure—particularly high concentrations of total hydrocarbons and methane—and increased uveitis risk (24). Other studies have suggested possible connections between fine particulate matter pollution and uveitis incidence (25), highlighting environmental pollution as a potential contributing factor. While these studies provide initial insights, significant knowledge gaps remain regarding the complex relationships between environmental factors and uveitis. Most previous research has examined linear relationships or short–term effects, without adequately accounting for potential non–linear associations and cumulative exposure impacts. Moreover, the interactive effects between temperature, humidity, and air pollutants remain poorly understood.

Shanghai, as China’s major economic center with a subtropical monsoon climate, experiences significant seasonal temperature variations and faces periodic air quality challenges. These distinct environmental characteristics, combined with the city’s comprehensive monitoring networks and advanced healthcare system, make it an ideal location to study potential associations between environmental factors and uveitis—an important cause of visual impairment worldwide. Understanding these relationships could inform targeted prevention strategies and contribute to reducing the disease burden in urban populations experiencing similar environmental conditions.

This study aims to examine potential associations between average temperature and uveitis outpatient visits in Shanghai, China, while accounting for non–linear relationships and lag effects. By employing a distributed lag non–linear model (DLNM) and stratifying analyses by demographic factors and environmental conditions, we seek to provide a more comprehensive understanding of how temperature might relate to uveitis occurrence patterns in a real–world setting.

## Methods

### Study region

Shanghai is located on China’s eastern coast (120°52′–122°12′E, 30°40′–31°53′N). The city experiences a subtropical monsoon climate with distinct seasons, characterized by hot, humid summers and cold, damp winters. As a major urban center, Shanghai faces both significant temperature fluctuations and air quality challenges, providing an opportunity to examine the potential impacts of these environmental factors on ocular inflammatory diseases.

### Data collection

We analyzed outpatient records of uveitis patients from January 2017 to December 2023 using the information system of the Eye, Ear, Nose and Throat Hospital of Fudan University, the largest ophthalmic specialty center in Shanghai. We collected de–identified data including gender, age, and residential area code. Uveitis diagnoses were identified using the International Classification of Diseases, 10th Revision (ICD–10) code H20.904. We applied the following exclusion criteria: (1) non–permanent Shanghai residents; (2) patients with infectious uveitis; and (3) return visits within 30 days of the initial consultation. It should be noted that this study did not differentiate between incident and chronic uveitis cases in our analysis. All uveitis outpatient visits were analyzed as a unified cohort. The rationale for this approach is threefold: (1) our research focus is on how environmental factors influence uveitis outpatient visits as a whole, rather than exploring specific disease subtypes or etiological mechanisms; (2) both new–onset and chronic cases seeking medical care may be similarly influenced by environmental triggers; (3) the exclusion criterion of return visits within 30 days of the initial consultation helps ensure that each visit represents a distinct clinical episode. After applying these criteria, 8,090 uveitis cases were included.

Meteorological data, including daily average temperature and relative humidity, were obtained from the Xihe Energy Meteorological Big Data Platform.^1^ Air pollutant data were acquired from the Shanghai Ecological Environment Bureau,^2^ including 24-h average concentrations of particulate matter less than 2.5 μm (PM_2.5_), particulate matter less than 10 μm (PM_10_), sulfur dioxide (SO_2_), nitrogen dioxide (NO_2_), carbon monoxide (CO), and 8-h monitoring concentrations of ozone (O_3_). These measurements represent daily average concentrations from fixed environmental monitoring stations throughout Shanghai (Supplementary Figure S1).

### Statistical analysis

We conducted descriptive analyses of uveitis outpatient visits, meteorological factors, and air pollutant concentrations, reporting means, standard deviations, minimums, medians, maximums, and interquartile ranges.

Given the non-normal distribution of meteorological and air pollutant data, we employed Spearman correlation analysis to explore associations between these variables and uveitis outpatient visits.

Since outpatient visits for uveitis are relatively rare events typically following a Poisson distribution, we employed a generalized linear model framework. To investigate potential delayed and cumulative effects of temperature exposure on uveitis risk while accounting for non-linear relationships, we utilized a distributed lag non-linear model with Poisson distribution as the link function (26). The model incorporated daily outpatient visits for uveitis as the dependent variable and daily average temperature as the independent variable.

To address multicollinearity, variables with *Spearman* correlation coefficients exceeding 0.7 were excluded (27). The model simultaneously adjusted for relative humidity, air pollutant concentrations, day-of-week effects, seasonal patterns, and long-term time trends. We also accounted for Chinese national holidays, which might influence healthcare-seeking behavior (28). The model was specified as:


$$\begin{array}{l} \log(Y_{t})=\alpha +\mathrm{cb}(\begin{array}{l} \text{Average}\;\\ {\text{temperature}}_{t},\mathrm{lag} \end{array})+\mathrm{ns}(\begin{array}{l} \text{Average}\;\\ \text{relative}\;\\ {\text{humidity}}_{t},\mathrm{df} \end{array})+\\ \mathrm{ns}(\mathrm{Air}\;{\text{pollutants}}_{t},\mathrm{df})+\mathrm{ns}({\text{Time}}_{t},\mathrm{df})+\\ \mathrm{as}.\text{factor}({\mathrm{DOW}}_{t})+\mathrm{as}.\text{factor}({\text{Holidays}}_{t}) \end{array}$$

Where *t* represents the date of observation; $Y_{t}$ denotes the daily visits for uveitis; α signifies the intercept; cb represents the matrix of average temperature constructed by the cross-basis function; ns is a natural cubic smooth spline function; $\mathrm{Air}\;{\text{pollutants}}_{t}$ indicates the daily average concentration of air pollutants including PM_2.5_, O_3_, SO_2_ and NO_2_; ${\text{Time}}_{t}$ represents the time trend, which is used to remove the long-term and seasonal trends that cannot be measured in time series data; df is the degree of freedom. ${\mathrm{DOW}}_{t}\;$ is the day of the week as a categorical variable; and ${\text{Holidays}}_{t}$ is a dummy variable for national holidays.

Based on the findings from previous studies and the Akaike Information Criterion (AIC), we determined a maximum lag period of 14 days to capture the delayed exposure effect of average temperature on uveitis risk (27) (Supplementary Table S1). According to the previous research results, the df of daily average relative humidity and average concentration of air pollutants was set to 3, and the df of ${\text{Time}}_{t}$ was set to 7 per year (28, 29).

For subgroup analyses, we stratified participants by gender, age (below vs. above 60 years), and median values of relative humidity and PM_2.5_ concentration (27). We used the median daily average temperature as the reference value and defined the first percentile (P1) as representing extremely low temperature (30).

We conducted sensitivity analyses by varying the df for time (8–11) and relative humidity (4–7) to test the robustness of our results. To account for potential confounding effects of the COVID-19 pandemic on healthcare access, we conducted sensitivity analyses by incorporating a binary pandemic indicator (1 = January 2020–December 2022; 0 = other periods) into the DLNM framework. This adjustment allowed us to evaluate the robustness of temperature-uveitis associations while controlling for pandemic-related healthcare disruptions.

All analyses were performed using R statistical software (version 4.2.3) with “dlnm,” “splines,” and “ggplot2” packages. Statistical significance was defined as two-sided *p* < 0.05.

## Results

### Data description

Between 2017 and 2023, we analyzed 8,090 uveitis outpatient visits, comprising 4,065 male and.

**Figure 1:**
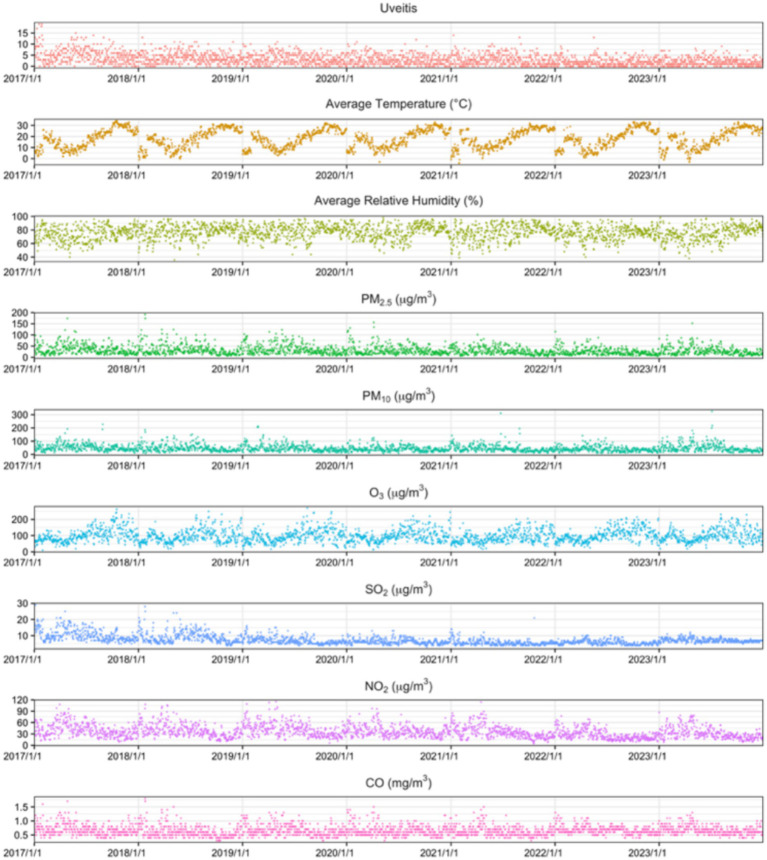
Daily changes in uveitis outpatient visits, meteorological factors, and air pollutants in Shanghai, 2017–2023.

Four thousand and twenty-five female patients. Daily outpatient visits ranged from 0 to 19 cases (mean: 3.17 cases/day). Daily average temperature ranged from −4°C to 34°C (mean: 17.85°C). Table 1 presents detailed descriptive statistics for outpatient visits, meteorological conditions, and air pollutant concentrations. Time series analysis revealed seasonal patterns in uveitis outpatient visits, daily average temperature, relative humidity, and air pollutant concentrations throughout the study period (Figure 1).

**Table 1:** Characteristics of outpatient visits for uveitis, meteorological conditions, and air pollutant concentrations from 2017 to 2023.

	Sum	Mean	SD	Min	P_25_	Median	P_75_	Max
Total visits	8,090	3.17	2.60	0	1	3	5	19
Male	4,065	1.59	1.59	0	0	1	2	13
Female	4,025	1.58	1.58	0	0	1	2	12
<60 years	5,562	2.18	1.97	0	1	2	3	17
≥60 years	2,528	0.99	1.18	0	0	1	2	7
AT (°C)		17.85	8.51	−4	10	18	25	34
RH (%)		76.3	10.62	36	70	77	84	98
PM_2.5_ (μg/m^3^)		31.22	20.78	3	17	26	40	191
PM_10_ (μg/m^3^)		46.17	26.97	7	29	39	57	325
O_3_ (μg/m^3^)		98.38	40.06	10	69	91	120	269
SO_2_ (μg/m^3^)		7.34	2.99	3	5	7	8	29
NO_2_ (μg/m^3^)		35.88	17.01	4	23	33	45	115
CO (mg/m^3^)		0.66	0.19	0.3	0.5	0.6	0.7	1.8

AT, average temperature; RH, relative humidity; PM_2.5_, particulate matter ≤ 2.5 microns in aerodynamic diameter; PM_10_, particulate matter ≤ 10 microns in aerodynamic diameter; O_3_, ozone; SO_2_, sulfur dioxide; NO_2_, nitrogen dioxide; CO, carbon monoxide; SD, standard deviation; P_25_, 25th percentile; P_75_, 75th percentile.

### Correlation analysis of environmental factors

**Figure 2:**
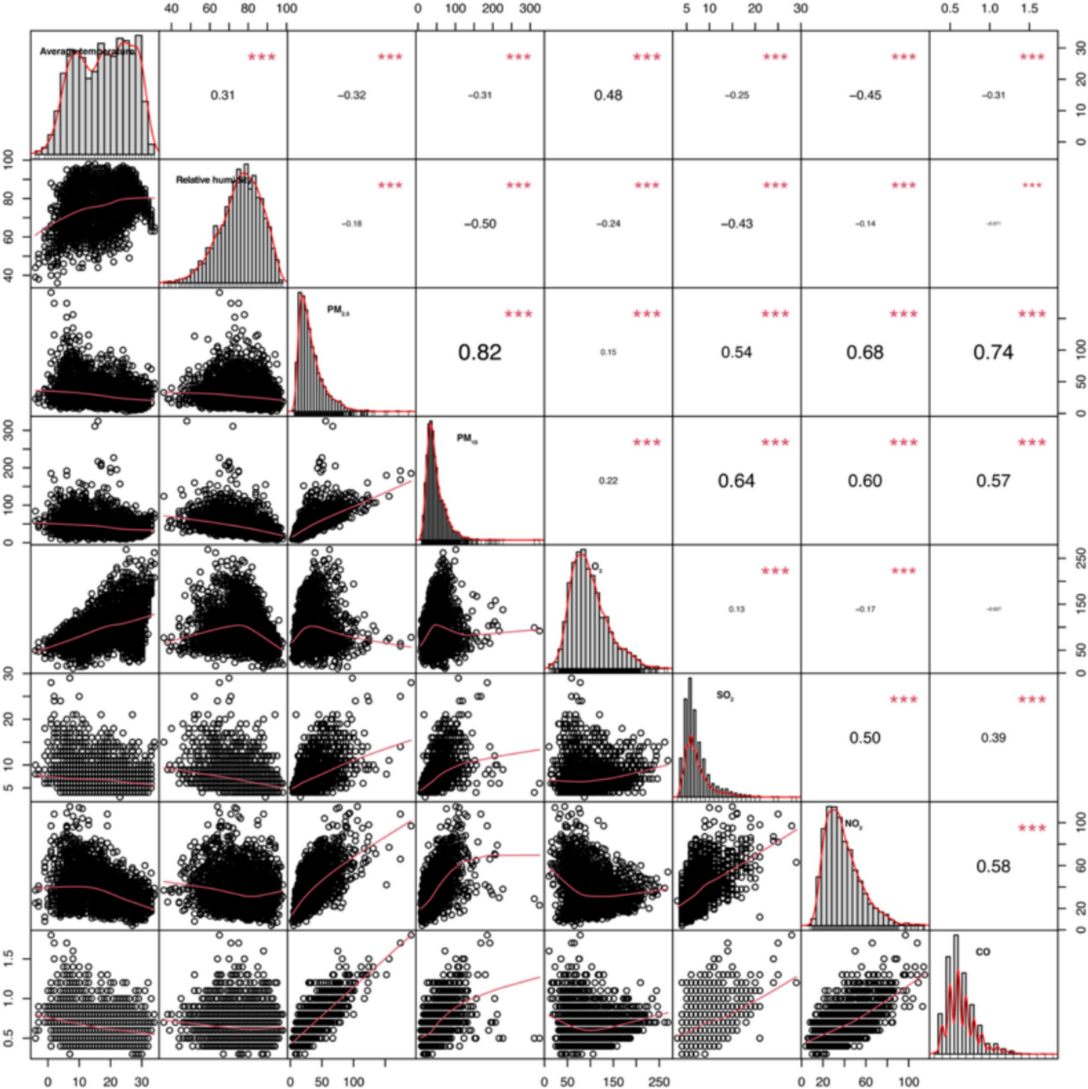
*Spearman*’s correlation coefficients and scatter plots of daily average temperature, daily relative humidity, and air pollutants from 2017 to 2023.

*Spearman* correlation analysis revealed significant associations between most meteorological factors and air pollutants. Strong positive correlations were observed between PM_2.5_ and PM_10_ (*r* = 0.82) and between PM_2.5_ and CO (*r* = 0.74). Daily average temperature showed positive correlations with relative humidity and O_3_ (*r* = 0.31 and 0.48, respectively) and negative correlations with other air pollutants (Figure 2).

### Association between temperature and uveitis outpatient visits

**Figure 3:**
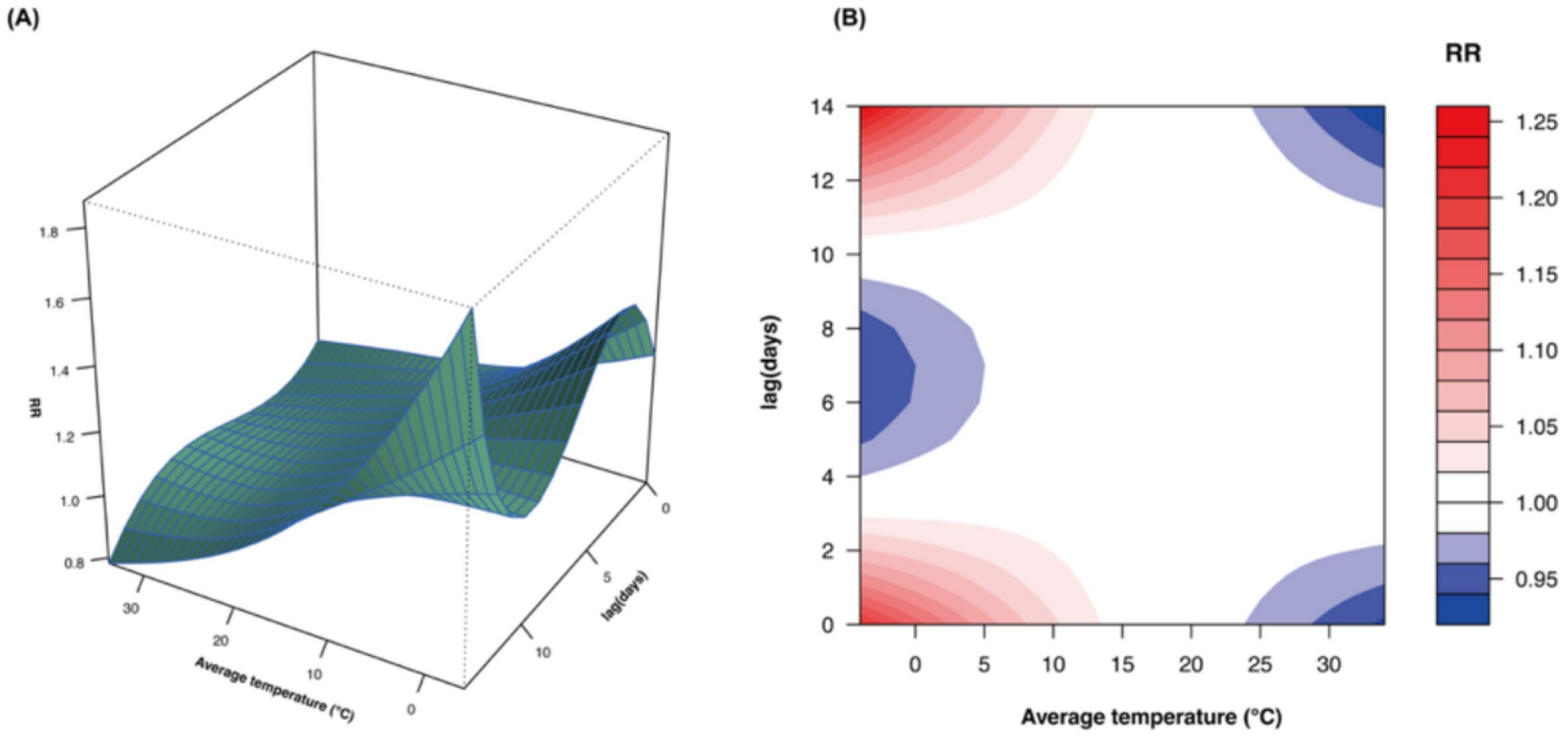
Cumulative **(A)** and single-day **(B)** lag effects of average temperature on outpatient visits for uveitis. RR, relative risk.

In the cumulative lag analysis (lag 0–3), we observed a significant non-linear association between lower temperatures and increased relative risk (RR) of uveitis outpatient visits. The strongest association occurred at lag 1 at −4°C (RR = 1.351, 95%CI: 1.069–1.706) (Figure 3A). For single-day lag effects, significant associations were observed at lag 0–1 and lag 12–14, with the highest RR at lag 14 at −4°C (RR = 1.257, 95%CI: 1.113–1.420) (Figure 3B).

**Figure 4:**
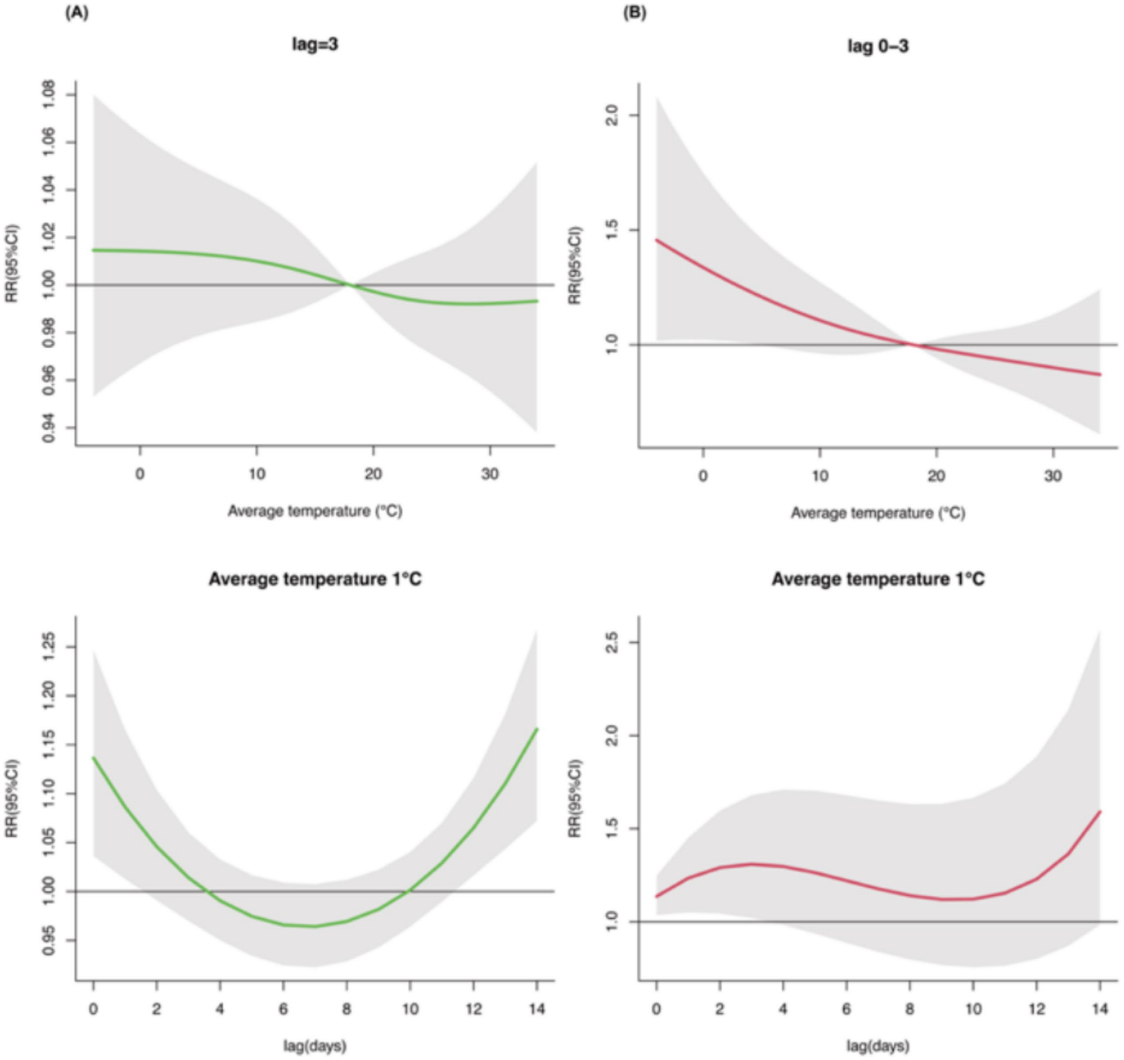
Single-day **(A)** and cumulative **(B)** lag effects of average temperature on outpatient visits for uveitis at lag3 or when exposed to extremely low temperatures (P1, 1°C).

At lag 3, we found no significant single-day effect, but a significant cumulative lag association for temperatures ranging from −4°C (RR = 1.455, 95%CI: 1.018–2.080) to 4°C (RR = 1.232, 95%CI: 1.006–1.509). When examining extremely low temperatures (P1, 1°C), significant single-day lag associations were observed at lag 0–1 and lag 12–14, with the strongest association at lag 14 (RR = 1.166, 95%CI: 1.072–1.267). The cumulative lag effect remained significant at lag 0–3, peaking at lag 3 (RR = 1.309, 95%CI: 1.021–1.680) (Figure 4).

### Demographic subgroup analyses

When stratifying by gender, we found that males showed significant cumulative lag associations between lower temperatures and increased uveitis outpatient visits during lag 0–1. At lag 1, temperatures from −4°C (RR = 1.382, 95%CI: 1.001–1.908) to 6°C (RR = 1.184, 95%CI: 1.006–1.394) were associated with increased risk, peaking at −4°C. No significant cumulative lag associations were observed in females (Supplementary Figure S2A). For single-day lag effects, males showed the strongest association at lag 14 at −4°C (RR = 1.580, 95%CI: 1.338–1.866), while females showed the strongest association at lag 7 at 34°C (RR = 1.124, 95%CI: 1.021–1.237) (Supplementary Figure S2B).

Age-stratified analysis revealed that individuals under 60 years showed increased risk of uveitis outpatient visits following exposure to lower temperatures during lag 0 (−4°C to 9°C) to lag 2 (−2°C to 6°C), with the strongest association at lag 2 at 1°C (RR = 1.316, 95%CI: 1.017–1.701). No significant cumulative lag associations were observed in those aged 60+ (Supplementary Figure S3A). For single-day lag effects, significant associations were found only in those under 60 at lag 0–1 and lag 12–14, with the strongest association at lag 14 at −4°C (RR = 1.301, 95%CI: 1.123–1.507) (Supplementary Figure S3B).

**Figure 5:**
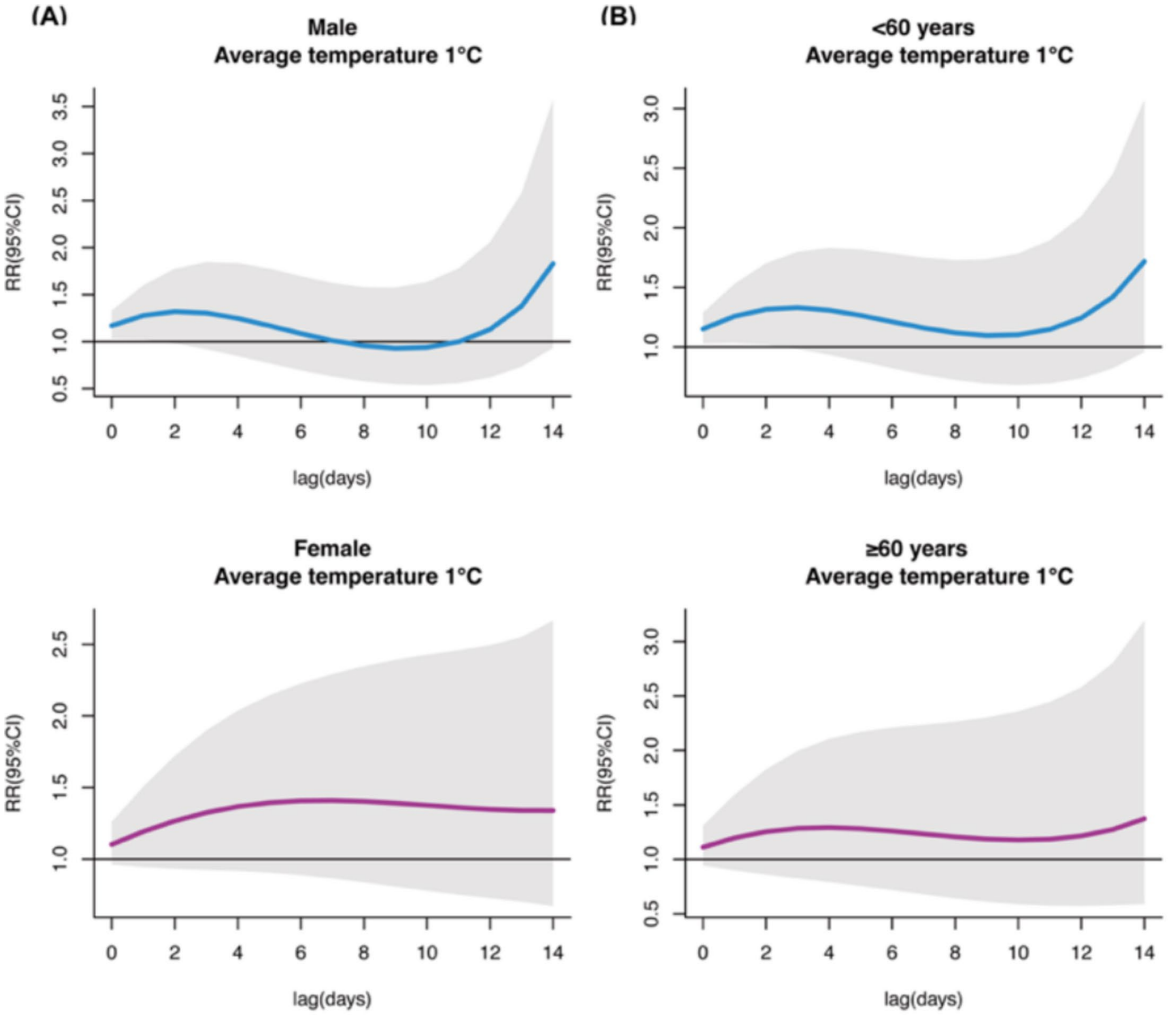
Cumulative lag effects of extreme low temperature (P1, 1°C) exposure on outpatient visits for uveitis in gender **(A)** and age **(B)** subgroups.

When examining extreme low temperatures (P1, 1°C), males and individuals under 60 showed increased risk of uveitis outpatient visits from lag 0–1 and lag 0–2, respectively, with peak associations at lag 1 (RR = 1.279, 95%CI: 1.021–1.600) and lag 2 (RR = 1.316, 95%CI: 1.017–1.701). No significant associations were observed in females or those aged 60 + at extreme low temperatures (Figure 5).

### Environmental modification analyses

When stratifying by humidity, under low relative humidity conditions, we observed significant associations between high temperatures (33–34°C) and increased uveitis outpatient visits at lag 14, with the strongest association at 34°C (RR = 2.625, 95%CI: 1.034–6.668). Under high humidity conditions, significant associations were observed between low temperatures (1–3°C) and increased visits during lag 9–11, peaking at 1°C at lag 11 (RR = 2.250, 95%CI: 1.000–5.060) (Supplementary Figure S4A). For single-day lag effects, under low humidity, significant associations were observed during lag 12–14 at 4°C, peaking at lag 14 (RR = 1.170, 95%CI: 1.043–1.316); under high humidity, significant associations were observed during lag 4–5 at low temperatures, peaking at 1°C at lag 5 (RR = 1.097, 95%CI: 1.005–1.197) (Supplementary Figure S4B).

In the PM_2.5_–stratified analysis, no significant associations were observed at lower PM_2.5_ levels. However, at higher PM_2.5_ levels, significant associations were observed between low temperatures (−3 to −2°C) and increased uveitis outpatient visits at lag 14, with the strongest association at −3°C (RR = 2.611, 95%CI: 1.028–6.631). Single-day lag analysis showed significant associations between low temperatures and increased visits from lag 9–12, peaking at −3°C at lag 12 (RR = 1.106, 95%CI: 1.012–1.208) (Supplementary Figure S5).

**Figure 6:**
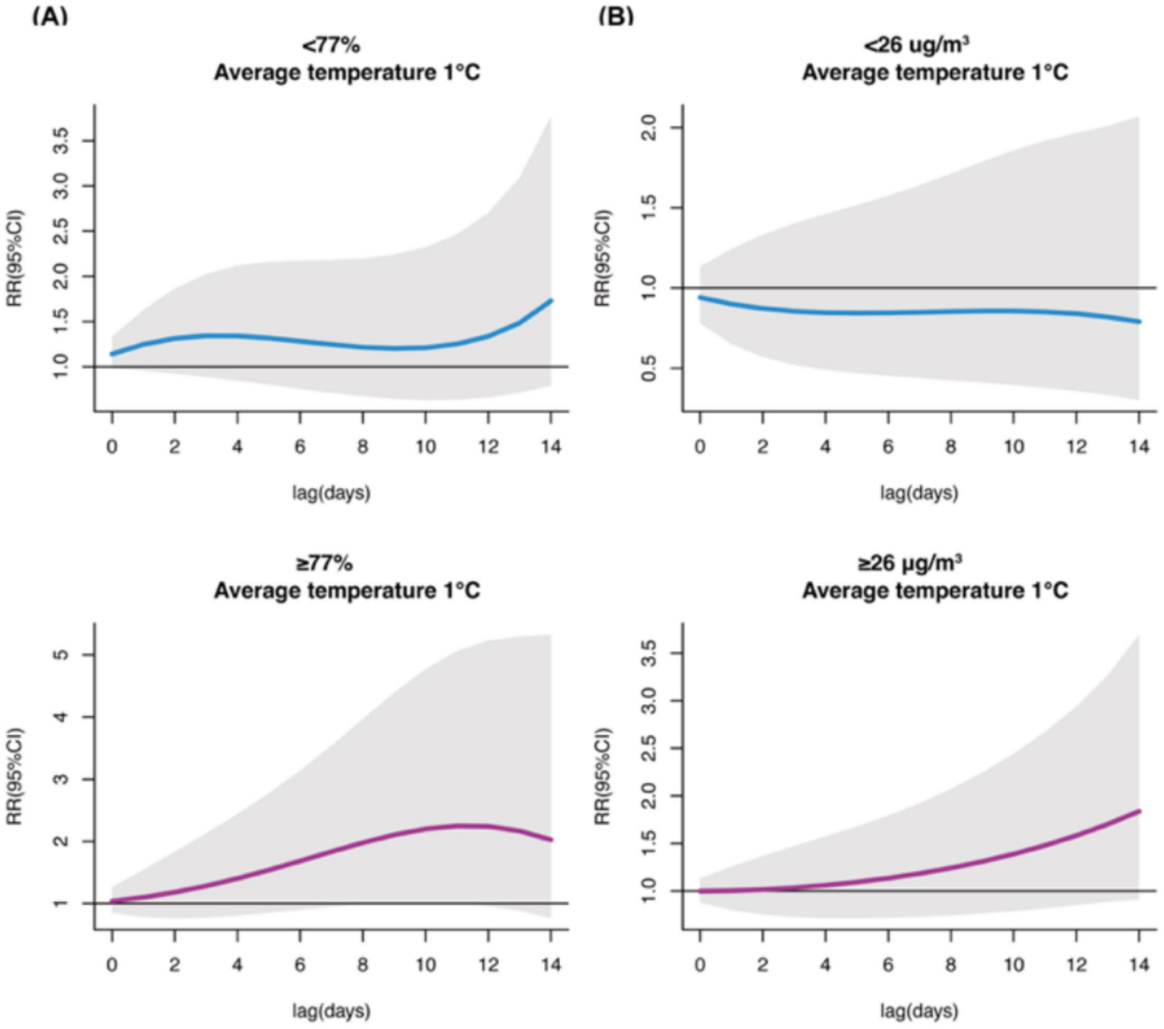
Cumulative lag effects of extreme low temperature (P1, 1°C) exposure on outpatient visits for uveitis in RH (%) **(A)** and PM_2.5_ **(B)** subgroups.

When examining extreme low temperatures (P1, 1°C) under high humidity conditions, we observed significant cumulative lag associations with increased uveitis outpatient visits, peaking at lag 11 (RR = 2.250, 95%CI: 1.000–5.060). No significant associations were observed at extreme low temperatures under low humidity conditions or in the PM_2.5_-stratified analysis (Figure 6).

### Sensitivity analysis

Our sensitivity analyses, varying the df for time and relative humidity, demonstrated that our findings remained consistent across different model specifications, supporting the robustness of our results (Supplementary Figure S6). Sensitivity analysis adjusting for the COVID-19 pandemic period showed minimal changes in effect estimates. Complete results of pandemic-adjusted models are presented in Supplementary Table S2.

## Discussion

The confirmed correlation between meteorological factors, air pollutants, and the incidence of autoimmune eye diseases such as Graves’ ophthalmopathy and allergic eye diseases extends to uveitis, another autoimmune eye disease (31). This study analyzes the characteristics of fluctuations in daily average temperature and outpatient visits for uveitis in Shanghai from 2017 to 2023, revealing the combined impact of daily average temperature, other climate factors, and air pollutants on the risk of outpatient visits for uveitis in Shanghai. These findings provide critical evidence for environmental health policy development and targeted public health interventions to protect vulnerable populations from climate-related ocular inflammatory diseases.

A study conducted by Gómez-Mariscal et al. investigated the impact of seasonal and environmental variations on the onset of uveitis, revealing a significant increase in its incidence during winter (23). An epidemiological investigation on uveitis in northeastern Italy reported a higher prevalence during cold months from November to February (with an average temperature below 8°C) compared to warmer months, while a Chinese study involving 2,000 uveitis cases also demonstrated that it is more commonly observed during the transitional period between autumn and winter, exhibiting a clear correlation with the month (22, 32). In our present study, we have observed similar phenomena as previous research where exposure to lower temperatures exhibited an accumulative-lag effect significantly increasing the risk of seeking medical attention for uveitis in early lag periods, with the strongest single-day lag effect occurring at 14 days. Therefore, we consider low temperature as an important environmental risk factor contributing to increased susceptibility to uveitis. This temperature-disease relationship has profound implications for healthcare resource allocation and seasonal disease preparedness strategies, particularly in regions experiencing extreme weather events due to climate change.

The incidence of uveitis exhibits demographic disparities within the population. Although uveitis can manifest at any age, it demonstrates a lower average age of onset. A previous global epidemiological survey on uveitis also indicated that 85.6% of patients were aged between 17 and 59, with an average age of 36.5 years (12, 13). Regarding gender distribution, the ratio of male to female patients with uveitis is comparable in developed countries (13). We observed similar characteristics in our study cohort comprising 8,090 cases, where individuals under the age of 60 constituted the majority (5,562 cases or 68.8%), and there was a balanced representation of male and female patients. The variation in uveitis incidence across different populations has prompted our curiosity regarding potential population-specific differences in the association between temperature and the likelihood of seeking medical treatment for uveitis. Participants were stratified by gender and age (below 60 years old, and 60 years old and above), and subsequent subgroup analysis revealed a higher propensity for increased risk of uveitis development among males and individuals below the age of 60 when exposed to low temperature environments. A study by Tan et al. on the relationship between the incidence of uveitis and temperature variations in mainland China found that males and individuals aged 20–50 were more significantly affected by temperature changes in terms of uveitis incidence (21). This is similar to our research findings, which indicate that males under 60 years old appear to be more susceptible to the impact of low temperatures. Shanghai has achieved universal health insurance coverage, which largely eliminates major barriers to healthcare access. We therefore consider that these findings may be related to the fact that males under 60 years are more likely to work outdoors and consequently experience greater exposure to temperature variations. These demographic vulnerability patterns are essential for developing targeted health protection strategies and informing occupational health policies for outdoor workers and vulnerable populations during extreme weather events. While we adjusted for basic demographic factors, residual confounding from unmeasured sociocultural variables (e.g., health literacy, care-seeking preferences) may persist. Future studies incorporating community-based behavioral data are warranted to further disentangle these effects.

Our study also found that both meteorological factors and air pollutants exhibit seasonal variations. Furthermore, the concentrations of other air pollutants show an increasing trend during the cold season, apart from ozone (O₃). Spearman correlation analysis with average temperature further confirms this trend by demonstrating a positive correlation between ozone concentration and average temperature (*r* = 0.48). This phenomenon can be attributed to the involvement of solar radiation in ozone formation, as its concentration tends to increase when solar radiation is abundant during warm seasons (33). Given the close relationship between meteorological factors and environmental pollutants, we were interested in investigating the differential impact of average temperature on outpatient visits for uveitis under different environmental conditions. Subgroup analysis reveals that patients are more likely to experience an increased risk of uveitis after exposure to low temperatures at higher relative humidity levels. Gómez-Mariscal et al. conducted a study on 731 episodes of uveitis in 594 patients from a hospital in Madrid and found that both winter seasons and the number of rainy days per month were correlated with the incidence of uveitis (23). This finding suggests that low temperatures and high humidity may serve as potential triggers for the onset of uveitis. Interestingly, our study further observed that exposure to extremely high temperatures, particularly under conditions of low relative humidity, increased the risk of uveitis outpatient visits. Cao et al. discovered that in regions with a subtropical monsoon humid climate, the hospitalization rate of uveitis may increase due to exposure to low relative humidity and high average temperatures. Similarly, we have observed similar phenomena in Shanghai, which also experiences a subtropical monsoon climate (27). Additionally, Tan et al. conducted a study in mainland China and reported that a 1°C increase in average temperature was associated with an increase of approximately 2 uveitis cases per 1,000 individuals (21). Collectively, these studies indicate that high temperatures may also represent a risk factor for uveitis. Although our study found that the overall risk of uveitis in Shanghai was primarily associated with low temperatures and high relative humidity, the impact of extremely high temperatures on uveitis risk cannot be disregarded in the context of global warming. This highlights the critical importance of acknowledging the disease burden associated with climate change and implementing comprehensive environmental health policies aimed at mitigating its effects on population health outcomes.

As the largest developing country and simultaneously the world’s top carbon emitter, China faces numerous challenges in the coordinated development of its economy, energy sector, and ecological environment. Among these challenges, the escalating issues of greenhouse gas emissions, predominantly carbon dioxide, and complex atmospheric pollution have become particularly prominent (32, 34). In response, China has actively implemented a national strategy to combat climate change, making significant commitments to carbon reduction on the international stage: achieving carbon peaking by 2030 and carbon neutrality by 2060. Furthermore, the Fifth Plenary Session of the 19th Central Committee of the Communist Party of China and the Central Economic Work Conference have explicitly emphasized the need to realize synergistic effects in emission and carbon reduction (35, 36). Our findings provide scientific evidence supporting these policy initiatives by demonstrating the direct health co-benefits of integrated climate and air quality management strategies.

PM_2.5_, a fine particulate matter within air pollutants, primarily originates from the combustion of kerosene, wood, and vehicular emissions (37). Despite notable improvements in China’s air quality following the implementation of emission and carbon reduction policies, the country still recorded the highest number of deaths attributed to PM_2.5_ pollution globally in 2019 (38). A study by Tan et al. revealed that a 10 μg/m (3) increase in PM_2.5_ concentration in mainland China was associated with an additional case of uveitis (25). In our study, we further stratified subgroups based on PM_2.5_ concentrations and observed that, under higher PM_2.5_ levels, low temperatures exerted a more pronounced cumulative-lag effect on uveitis-related outpatient visits, thereby increasing the risk for patients. Additionally, the concentration of PM_2.5_ in China tends to rise during colder seasons due to increased usage of fuels such as coal and wood, highlighting the necessity for heightened attention to PM_2.5_ management in order to mitigate the impact of low temperatures on uveitis incidence. These findings underscore the urgent need for comprehensive air quality management policies that address both seasonal heating practices and long-term energy transition strategies to protect vulnerable populations from compound environmental health risks.

The demonstrated synergistic effects between temperature extremes and PM_2.5_ pollution provide compelling evidence for integrated environmental health approaches that address climate and air quality simultaneously. These findings support China’s carbon neutrality commitments by demonstrating direct health co-benefits of clean energy policies, particularly for winter heating systems where both cold exposure and particulate matter concentrations peak. Healthcare systems can utilize our identified temporal patterns and demographic vulnerability profiles to optimize resource allocation during high-risk periods, while clinical practitioners may integrate weather forecasting into patient counseling for susceptible populations, especially males under 60 years and outdoor workers who face enhanced occupational exposure risks.

Our findings suggest three practical measures: (1) protective guidelines for outdoor workers during temperature extremes, (2) early warning systems combining meteorological and air quality data, and (3) environment-aware clinical follow-up schedules. However, several limitations should be acknowledged in this study. First, as a hospital-based retrospective analysis, we could not fully control for all potential confounding factors, such as individual behaviors or occupational exposures, thus precluding causal inferences between temperature and uveitis risk. Second, although the sample size was substantial, data were derived solely from a tertiary ophthalmic center in Shanghai, which may limit generalizability to other regions. Third, while we employed DLNM, meteorological and pollutant data from fixed monitoring stations might not precisely reflect individual exposure levels. Fourth, while our sensitivity analysis incorporating a binary pandemic indicator showed consistent results, residual confounding from unmeasured pandemic effects (e.g., delayed diagnosis due to healthcare avoidance) may persist. However, the stability of effect estimates across pandemic and non-pandemic periods strengthens confidence in our primary findings regarding temperature-uveitis associations. Finally, other environmental determinants were not incorporated. Future multicenter prospective studies incorporating personalized exposure assessments are warranted to validate these findings.

In conclusion, our study highlights young males and low temperatures as key risk factors for uveitis, while demonstrating that extremely high temperatures in low humidity environments also pose significant health risks. Moreover, the amplified effects of high PM_2.5_ concentrations combined with cold temperatures emphasize the critical need for refined, integrated air pollution control policies in China to protect public health. These findings provide robust scientific evidence supporting coordinated environmental health policies that address climate change mitigation, air quality improvement, and population health protection simultaneously, ultimately contributing to more resilient and healthy urban communities.

## Data Availability

The original contributions presented in the study are included in the article/Supplementary material, further inquiries can be directed to the corresponding authors.

## Footnotes

^1^ https://xihe-energy.com/

^2^ https://sthj.sh.gov.cn/
