# Supplementary material for: Climate change and ocular health: temperature-pollution synergies amplify uveitis burden
Source: Front Public Health. 2025 Jul 25;13:1650255. doi: 10.3389/fpubh.2025.1650255 (PMC12331684; doi:10.3389/fpubh.2025.1650255)
Supplement: Supplementary file 1 [file Data_Sheet_1.pdf]

Supplements

Supplementary Table. 1 The AIC values of the model under different lag days

**Lag**	**0**	**1**	**2**	**3**	**4**	**5**	**6**	**7**	**8**	**9**	**10**	**11**	**12**	**13**	**14**
**AIC**	10068	10062	10059	10055	10054	10048	10042	10034	10031	10027	10022	10016	10010	10001	**9993.8**

Lag, Days of lag; AIC, Akaike Information Criterion

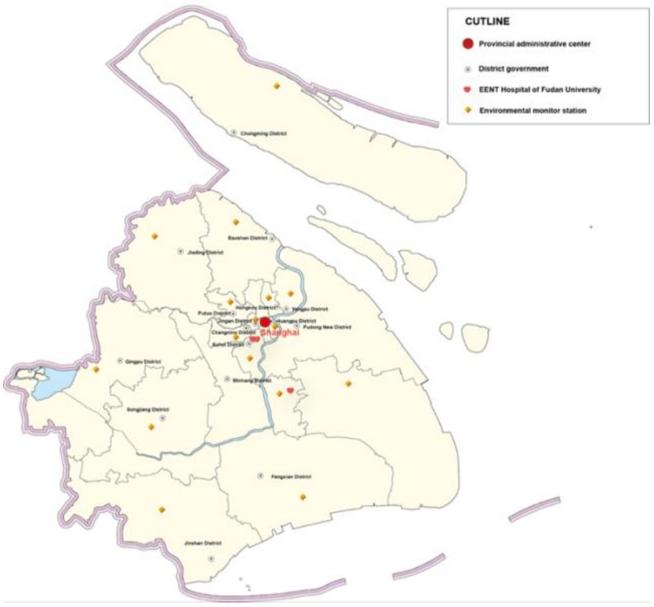

Supplementary Fig. 1 The location of the EENT Hospital of Fudan University and the Environmental Monitoring Station.

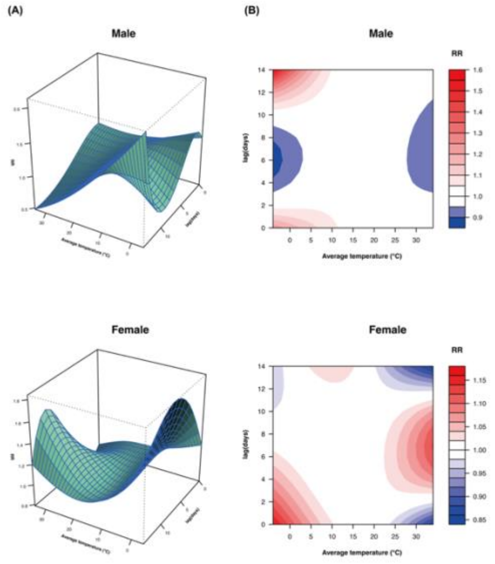

Supplementary Fig. 2 Cumulative **(A)** and single-Day **(B)** lag effects of average temperature on outpatient visits for uveitis in different gender subgroups. RR, relative risk

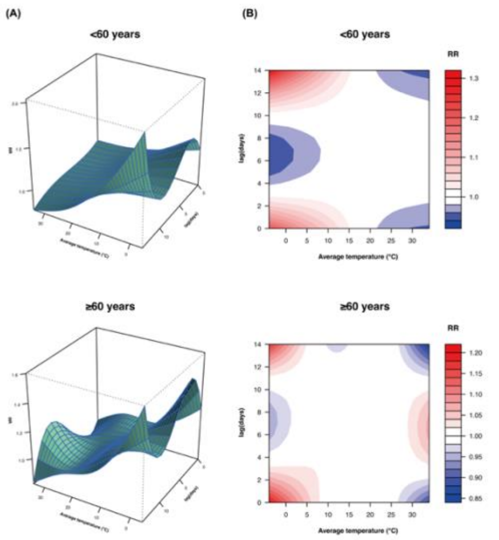

Supplementary Fig. 3 Cumulative **(A)** and single-Day **(B)** lag effects of average temperature on outpatient visits for uveitis in different age subgroups. RR, relative risk

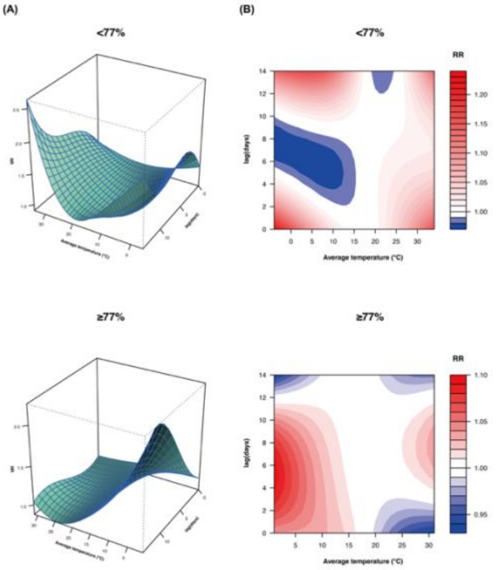

Supplementary Fig. 4 Cumulative **(A)** and single-Day **(B)** lag effects of average temperature on outpatient visits for uveitis in different RH(%) subgroups. RR, relative risk

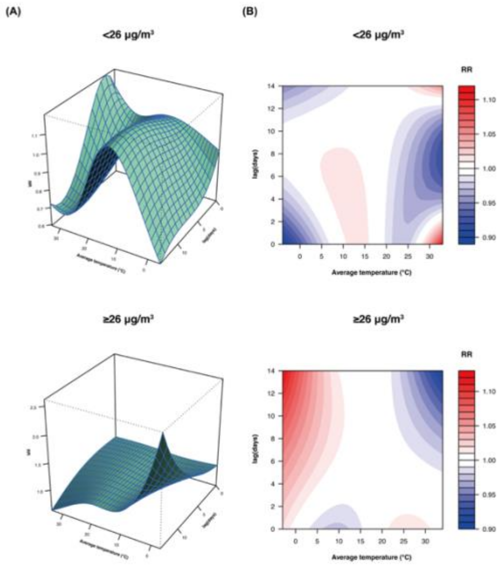

Supplementary Fig. 5 Cumulative **(A)** and single-Day **(B)** lag effects of average temperature on outpatient visits for uveitis in different $PM_{2.5}$ subgroups. RR, relative risk

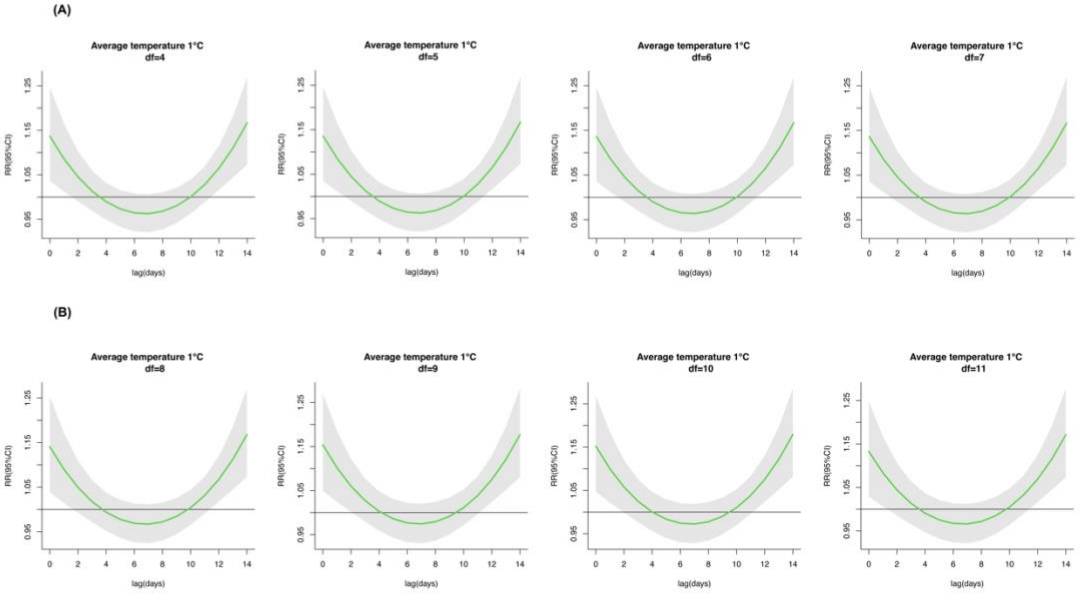

Supplementary Fig. 6 The single-day association between extreme low average temperature (1th, 1℃) and outpatient admissions for uveitis when altering the df (4-7 *df*) for average relative humidity **(A)** and the df (8-11 *df* /year) for time **(B)**.

Supplementary Table. 2 Comparison of temperature-uveitis associations between primary and pandemic-adjusted models

Temperature Condition	Primary Model RR (95% CI)	Pandemic-adjusted RR (95% CI)
-4°C at lag 1	1.351(1.069-1.706)	1.365(1.080-1.725)
34°C at low humidity	2.625(1.034-6.668)	2.600(1.024-6.606)
